# Increased Blood Levels of Growth Factors, Proinflammatory Cytokines, and Th17 Cytokines in Patients with Newly Diagnosed Type 1 Diabetes

**DOI:** 10.1371/journal.pone.0142976

**Published:** 2015-12-04

**Authors:** Kristi Alnek, Kalle Kisand, Kaire Heilman, Aleksandr Peet, Karin Varik, Raivo Uibo

**Affiliations:** 1 Department of Immunology, Institute of Bio- and Translational Medicine, University of Tartu, Estonia; 2 Tallinn Children’s Hospital, Tallinn, Estonia; 3 Department of Pediatrics, University of Tartu, Tartu, Estonia; 4 Children’s Clinic of Tartu University Hospital, Tartu, Estonia; 5 Surgery Clinic of Tartu University Hospital, Tartu, Estonia; La Jolla Institute for Allergy and Immunology, UNITED STATES

## Abstract

The production of several cytokines could be dysregulated in type 1 diabetes (T1D). In particular, the activation of T helper (Th) type 1 (Th_1_) cells has been proposed to underlie the autoimmune pathogenesis of the disease, although roles for inflammatory processes and the Th_17_ pathway have also been shown. Nevertheless, despite evidence for the role of cytokines before and at the onset of T1D, the corresponding findings are inconsistent across studies. Moreover, conflicting data exist regarding the blood cytokine levels in T1D patients. The current study was performed to investigate genetic and autoantibody markers in association with the peripheral blood cytokine profiles by xMap multiplex technology in newly diagnosed young T1D patients and age-matched healthy controls. The onset of young-age T1D was characterized by the upregulation of growth factors, including granulocyte macrophage-colony stimulating factor (GM-CSF) and interleukin (IL)-7, the proinflammatory cytokine IL-1β (but not IL-6 or tumor necrosis factor [TNF]-α), Th_17_ cytokines, and the regulatory cytokines IL-10 and IL-27. Ketoacidosis and autoantibodies (anti-IA-2 and -ZnT8), but not human leukocyte antigen (HLA) genotype, influenced the blood cytokine levels. These findings broaden the current understanding of the dysregulation of systemic levels of several key cytokines at the young-age onset of T1D and provide a further basis for the development of novel immunoregulatory treatments in this disease.

## Introduction

As cell-signaling molecules, cytokines play integral roles in the development and activation of immune cells. Much attention has been devoted to exploring their role in autoimmune diseases, including type 1 diabetes (T1D). Several disease-promoting cytokines and chemokines are dysregulated in the blood of prediabetic and diabetic patients [1,2,3]. Therefore, cytokines may serve as additional markers of T1D. Additionally, cytokines may provide valuable information about the pathogenic pathways and the regulation of disease processes, leading to the development of immunotherapeutic strategies (e.g., targeted neutralization of cytokines with monoclonal antibodies).

An imbalance towards T helper (Th) type 1 (Th_1_) cells and dysregulation of regulatory T (T_reg_) cells have been proposed to underlie the pathogenesis of T1D. Recently, the involvement of third effector pathway, that of Th_17_ cells, was shown to be connected with the pathogenesis of T1D and the destruction of pancreatic β-cells [4,5]. Despite evidence for the role of cytokines before and at the onset of T1D, the findings are inconsistent across studies, and conflicting data exist regarding the blood cytokine levels in patients with T1D. Plasma levels of proinflammatory and Th_1_ cytokines, such as interleukin (IL)-1β, IL-2, IL-6, IL-12, tumor necrosis factor (TNF)-α, and interferon (IFN)-γ, may be upregulated in patients with T1D [1,6–9]. However, other studies reported no difference in [2,10] or even reduced production of Th_1_-associated cytokines at the onset of T1D [3,11–13]. Moreover, metabolic imbalance (e.g., ketoacidosis, hyperglycemia, oxidative stress), disease duration, as well as the patient’s gender and age may influence the serum cytokine level in T1D [14,15].

The purpose of the present study was to investigate the peripheral blood immunoregulatory milieu (cytokine profiles of Th_1_, Th_2_, and Th_17_) in association of genetic and autoantibody markers in newly diagnosed young T1D patients and age-matched healthy controls. Stringent storage conditions (temperature < -70°C) and avoidance of repeated thawing of plasma or serum are needed for the correct measurement of cytokine levels in peripheral blood. Ideally, a multiplex platform should be used for the simultaneous detection of different cytokines. Therefore, in this study, the xMap multiplex technology was applied to assess 20 different cytokines simultaneously by two commercially available Luminex kits.

## Materials and Methods

### Study population

This study included 36 newly diagnosed young T1D patients (median age 10.5 years; interquartile range [IQR] 5.2–12.9 years; 17 boys/19 girls) and 20 controls (median age 14.6 years; IQR 6.7–20.3 years; 8 males/12 females). All patients were recruited from November 2008 to October 2011 at Tartu University Hospital and Tallinn Children’s Hospital. Diagnostic criteria for T1D were based on the classification of the Expert Committee on the Diagnosis and Classification of Diabetes Mellitus [16]. Data about concomitant autoimmune diseases (autoimmune thyroiditis, Graves' and Addison's diseases, celiac disease, vitiligo, autoimmune liver and rheumatic diseases, multiple sclerosis) and other diseases were available. No autoimmune diseases were recorded except two persons with autoimmune thyroiditis and one without diagnosis but with high anti-TPO antibodies in T1D group. None of individuals in studied population has a history of infections during last month. Peripheral blood was obtained less than 1 week after diagnosis. All T1D patients were on insulin treatment during the blood collection period (mean 0.72, range 0.15–1 U/kg of day). C-peptide values in T1D group were 0.132±0.077 nmol/L (mean±SD). The control group consisted of young healthy blood donors and children who visited Tartu University Hospital with minor surgical problems. Subjects in the control group did not have diabetes or abnormal fasting blood glucose levels, and there was no suspicion of any inflammatory process in these individuals.

All blood samples were collected in the morning before meals and EDTA-treated plasma from the blood was aliquoted and stored at -80°C. Samples did not go through additional freeze-thaw cycles before analysis. The study was approved by the Research Ethics Committee of the University of Tartu (protocols 163/T-6 from 24.09.2007 and 179/M-29 from 16.02.2009). All patients, their parents, and/or their guardians signed a written consent form before participation.

### Autoantibodies and human leukocyte antigen (HLA) genotyping

In all patients and controls, the presence of the main diabetes-associated antibodies and HLA class II alleles was determined. Autoantibodies against 65-kDa glutamic acid decarboxylase (GADA), protein tyrosine phosphatase (IA2A), and zinc transporter 8 (ZnT8A) were measured by commercial ELISA kits (RSR Ltd., Cardiff, UK), in accordance with the manufacturer’s protocol. The cut-off levels were ≥5 U/ml for GADA, ≥15 U/ml for IA2A and ZnT8A. These tests are performed routinely with specificity of 96–99% and sensitivity of 66–74%, as confirmed by the Islet Autoantibody Standardization Program (IASP) in 2012.

For determination of HLA DQA1–DQB1 genotypes and DRB1*04 subtypes, polymerase chain reaction (PCR)-based lanthanide-labeled oligonucleotide hybridization and time-resolved fluorometry were used [17,18]. Combinations of HLA DRB1-DQA1-DQB1 alleles were divided into five groups on the basis of risk for T1D development: high-, moderate-, slightly increased-, neutral-, and decreased-risk groups [18]. General medical information, autoantibodies, and HLA data for the study groups are presented in Table 1.

**Table 1:** Medical data, autoantibodies, and HLA haplotype frequencies in T1D patients and controls.

Characteristics	Controls (n = 20)	T1D patients (n = 36)
Ketoacidosis	-	44% (16/36)
Autoantibodies:		
GADA	10% (2/20^#^)	89% (32/36)
IA2A	0% (0/20)	69% (25/36)
ZnT8A	5% (1/20^#^)	78% (28/36)
HLA haplotypes:		
High risk^a^	0% (0/20)	25% (9/36)
Moderate risk^b^	10% (2/20)	28% (10/36)
Slightly increased risk^c^	0% (0/20)	8% (3/36)
Neutral risk^d^	25% (5/20)	19% (7/36)
Decreased risk^e^	65% (13/20)	19% (7/36)

^#^ Controls with a low level of GADA or ZnT8.

^a^ Heterozygosity for the two risk-associated haplotypes DRB1*0401/2/4/5/8-DQA1*03-DQB1*0302/4 and [DRB1*03]-DQA1*05-DQB1*02.

^b^ Above risk haplotypes as homozygous or DRB1*0401/2/4/5/8-DQA1*03-DQB1*0302/4 combined with a neutral haplotype, or the [DRB1*03]-DQA1*05-DQB1*02/[DRB1*09]-DQA1*03-DQB1*03 genotype.

^c^ [DRB1*03]-DQA1*05-DQB1*02 with a neutral haplotype or theDRB1*0401/2/5/8-DQA1*03-DQB1*0302/4/[DRB1*1301]-[DQA1*01]-DQB1*0603 genotype.

^d^ Genotypes where a risk haplotype is combined with a protective one(DRB1*15-[DQA1*01]-DQB1*0602, [DRB1*11/12/13]-DQA1*05-DQB1*0301, [DRB1*14]-[DQA1*01]-DQB1*0503, DRB1*07-DQA1*0201-DQB1*0303, DRB1*0403-[DQA1*03]-DQB1*0302/4 and [DRB1*1301]-[DQA1*01]-DQB1*0603 (except the combination in c)) or combinations of two neutral haplotypes.

^e^ Combinations of two protective haplotypes or a protective haplotype associated with a neutral one.

### Cytokines

Cytokine levels in EDTA-treated plasma from controls and T1D patients were measured by the xMAP Technology on Luminex 200 (Luminex Corp., Austin, TX). Levels of 20 different cytokines were determined with the Milliplex MAP High Sensitivity Human Cytokine kit (n = 20 controls, n = 34 T1D patients) and the Milliplex MAP Human Th17 Magnetic Bead Panel kit (n = 20 controls, n = 36 T1D patients; Millipore Corp., Billerica, MA) (Table 2). Cytokine levels were analyzed in accordance with the manufacturer’s protocol, with levels below the detection limit being imputed as 10% less than the minimum detectable concentration limit, as calculated by the manufacturer’s protocol.

**Table 2:** Minimal detectable concentrations and percentage rates of detection for investigated cytokines.

Cytokine	Kit name	Minimal detectable concentration^*^	Relative assay sensitivity ^**^
GM-CSF	High Sensitivity Human Cytokine	0.46 pg/mL	100%
IL-7	High Sensitivity Human Cytokine	0.12 pg/mL	100%
IL-1β	High Sensitivity Human Cytokine	0.06 pg/mL	100%
IL-6	Human Th17 Magnetic Bead Panel	1.7 pg/mL	96%
IL-6	High Sensitivity Human Cytokine	0.10 pg/mL	100%
TNF-ɑ	High Sensitivity Human Cytokine	0.05 pg/mL	100%
IL-8	High Sensitivity Human Cytokine	0.11 pg/mL	100%
IL-12 p70	High Sensitivity Human Cytokine	0.11 pg/mL	100%
IFN-γ	Human Th17 Magnetic Bead Panel	1.8 pg/mL	100%
IFN-γ	High Sensitivity Human Cytokine	0.29 pg/mL	100%
IL-2	High Sensitivity Human Cytokine	0.16 pg/mL	100%
IL-4	High Sensitivity Human Cytokine	0.13 pg/mL	100%
IL-5	High Sensitivity Human Cytokine	0.01 pg/mL	100%
IL-13	High Sensitivity Human Cytokine	0.48 pg/mL	89%
IL-17A	Human Th17 Magnetic Bead Panel	2.1 pg/mL	100%
IL-17E	Human Th17 Magnetic Bead Panel	0.099 ng/mL	98%
IL-17F	Human Th17 Magnetic Bead Panel	0.009 ng/mL	95%
IL-21	Human Th17 Magnetic Bead Panel	2 pg/mL	100%
IL-22	Human Th17 Magnetic Bead Panel	0.021 ng/mL	98%
IL-23	Human Th17 Magnetic Bead Panel	0.098 ng/mL	100%
IL-27	Human Th17 Magnetic Bead Panel	0.063 ng/mL	100%
IL-10	Human Th17 Magnetic Bead Panel	0.3 ng/mL	91%
IL-10	High Sensitivity Human Cytokine	0.15 pg/mL	100%

^*^ Calculated by the Millipore kit protocols.

^**^calculated as the frequency of detectable values in the plasma samples (n = 56); all extrapolated sample values were considered undetectable.

### Statistical analysis

The R version 3.0.1 language and environment (Free Software Foundation, Boston, MA) and GraphPad Prism 5 (GraphPad Software, La Jolla, CA) software packages were used for statistical analyses and figure preparation. For descriptive statistics, medians and IQRs are reported. As the concentrations of cytokines do not assume a normal distribution, the non-parametric, pairwise Spearman’s rank correlation was used to assess the correlation between cytokine levels. The Mann–Whitney *U*-test (two-tailed) was used to compare the characteristics of the two study groups and Kruskal-Wallis Rank Sum Test for more than two groups. A p-value less than or equal to 0.0025 after Bonferroni correction was considered statistically significant.

## Results

### Correlation of cytokine concentrations

Three cytokines, IL-6, IFN-γ, and IL-10, were measured simultaneously with High Sensitivity Human Cytokine kit and Human Th17 Magnetic Bead Panel kit. No strong correlations (*rho* < 0.7) were found between the concentrations of these cytokines when measured by the two assay kits in the whole study group, but correlation was statistically significant (Spearman’s rank correlation test: *rho* = 0.45, p = 0.0005 for IL-6 measured by Th17 kit versus High Sensitivity kits; *rho* = 0.60, p = 1.4 × 10^−6^ for IFN-γ measured by Th17 kit versus High Sensitivity kits, and *rho* = 0.56, p = 9.9 × 10^−6^ for IL-10 measured by Th17 kit versus High Sensitivity kits). The High Sensitivity Human Cytokine kit provided higher percentages of the detected cytokines (Table 2) and was used in the subsequent experiments.

### Relationship between cytokine levels in T1D patients and controls

In T1D patients, 20 cytokines from different functional groups were mutually correlated with each other. For example, granulocyte-macrophage colony-stimulating factor (GM-CSF) showed strong correlations (*rho* > 0.7) with the lymphoid hematopoietic growth factor IL-7 and with the T-cell growth factors IFN-γ and IL-2. An important mediator of the inflammatory response, IL-1β, showed strong correlations with several growth factors, namely GM-CSF, IL-7, IFN-γ, and IL-2. Interestingly, the well-known proinflammatory cytokines, IL-6, TNF-α, and IL-8, demonstrated weak correlations with other investigated cytokines, including IL-1β. As expected, IL-17A, IL-17E, IL-17F, IL-21, IL-22, and IL-23 were correlated strongly with each other, forming a large Th_17_ cytokine cluster. IL-27, which prevent excessive T cell activity and limit pro-inflammatory cytokine production, was also very strongly correlated with the Th_17_ cytokine cluster in T1D (Table 3, part A).

**Table 3:** The correlation matrix of peripheral blood cytokine levels in T1D patients (part A) and control group (part B).

**A**																				
**T1D**	GM-CSF	**IL-7**	**IL-1β**	IL-6	**TNF-ɑ**	**IL-8**	**IL-12**	**IFN-γ**	**IL-2**	**IL-4**	**IL-5**	**IL-13**	**IL-17A**	**IL-17E**	**IL-17F**	**IL-21**	**IL-22**	**IL-23**	**IL-27**	**IL-10**
**GM-CSF**																				
**IL-7**	**0.79**																			
**IL-1β**	**0.97**	**0.78**																		
**IL-6**																				
**TNF-ɑ**																				
**IL-8**				**0.52**	**0.50**															
**IL-12**	**0.55**	**0.58**	**0.56**	0.41		0.35														
**IFN-γ**	**0.78**	**0.66**	**0.73**				**0.59**													
**IL-2**	**0.82**	**0.61**	**0.82**				**0.50**	**0.71**												
**IL-4**	0.38	0.36	0.41	0.41		0.44	**0.55**	**0.55**	0.41											
**IL-5**		0.39	0.37		0.40	**0.54**	**0.52**	0.41	0.40	0.47										
**IL-13**	0.35	**0.60**	0.42	0.49		0.49	0.49	0.36	0.37	**0.69**	**0.61**									
**IL-17A**	**0.60**	0.47	**0.59**				**0.53**	**0.53**	**0.64**	0.42	0.41	0.46								
**IL-17E**	**0.51**		0.50				**0.52**	0.39	**0.55**	0.41			**0.91**							
**IL-17F**							0.34		0.37		0.47		**0.79**	**0.85**						
**IL-21**	0.49		0.49				0.39	0.34	**0.55**	0.35		0.37	**0.87**	**0.84**	**0.75**					
**IL-22**	**0.50**	0.35	**0.51**				0.50	0.44	**0.58**	0.49	0.40	0.49	**0.90**	**0.95**	**0.86**	**0.85**				
**IL-23**	**0.54**		**0.53**				**0.51**	0.44	**0.63**	0.44	0.37	0.45	**0.94**	**0.96**	**0.86**	**0.89**	**0.96**			
**IL-27**	0.42	0.39	0.41				0.44	0.35	**0.56**	0.40	0.40	0.42	**0.88**	**0.91**	**0.87**	**0.83**	**0.92**	**0.94**		
**IL-10**	0.45	0.39	0.44	**0.52**	0.35	**0.61**	**0.50**	**0.51**		0.49	0.49	**0.56**	0.40				0.34		0.39	
**B**																				
**Controls**	**GM-CSF**	**IL-7**	**IL-1β**	**IL-6**	**TNF-ɑ**	**IL-8**	**IL-12**	**IFN-γ**	**IL-2**	**IL-4**	**IL-5**	**IL-13**	**IL-17A**	**IL-17E**	**IL-17F**	**IL-21**	**IL-22**	**IL-23**	**IL-27**	**IL-10**
**GM-CSF**																				
**IL-7**	**0.77**																			
**IL-1β**	**0.93**	**0.69**																		
**IL-6**	0.44	**0.76**																		
**TNF-ɑ**	**0.66**	0.47	0.58																	
**IL-8**	**0.65**	0.61	0.64	0.58																
**IL-12**		0.58		**0.72**																
**IFN-γ**	**0.70**	**0.65**	0.63	0.47	**0.69**															
**IL-2**	**0.81**	**0.85**	**0.76**	**0.72**	0.52	0.64	0.54	**0.64**												
**IL-4**	0.62	**0.69**	0.48	0.62	0.56	**0.68**	0.46	0.55	0.61											
**IL-5**	0.46	0.56			0.59			0.48		0.44										
**IL-13**		0.52		0.52		0.54	0.54		0.48	**0.73**										
**IL-17A**	0.59	0.62	0.45	0.60	0.48	0.47		0.49	0.56	**0.79**	0.48	0.61								
**IL-17E**						0.54				0.46		0.60	**0.80**							
**IL-17F**						**0.77**						**0.64**	**0.66**	**0.94**						
**IL-21**	0.59	0.57		0.54	0.44	**0.71**		0.44	0.55	**0.70**		0.61	**0.96**	**0.66**	**0.72**					
**IL-22**						0.55				0.58		**0.73**	**0.77**	**0.91**	**0.95**	**0.81**				
**IL-23**						0.61				0.59		**0.70**	**0.83**	**0.88**	**0.89**	**0.87**	**0.96**			
**IL-27**						0.54				0.45		**0.65**	0.64	**0.84**	**0.94**	**0.66**	**0.91**	**0.88**		
**IL-10**	0.50	0.63	0.52		0.60	0.55		0.63	0.52		0.63	0.55	0.47			0.49		0.52		

* Pairwise Spearman’s rank correlation coefficient (*rho*); results with p ≤ 0.0025 are shown in bold; results with p > 0.05 are not shown.

In the control group, strong correlations were detected among GM-CSF, IL-7, IFN-γ, and IL-1β. IL-7 was strongly correlated with inflammatory marker IL-6 and T cell growth factor IL-2, which, in turn, were correlated with each other. There was also a strong correlation between IL-2 and IL-1β. In addition, IL-6 was correlated with IL-12. The neutrophil chemotactic factor IL-8 revealed a strong correlation with Th_17_ lymphocyte cytokines IL-17F and IL-21. IL-4 showed a strong correlation with another Th_2_ cytokine, IL-13, and with the Th_17_ cytokines IL-17A and IL-21. Moreover, IL-13 was correlated strongly with IL-22 and IL-7. Most of the Th_17_ cytokines were correlated strongly with each other (Table 3, part B).

### Age-, gender- and seasonal-dependency of cytokine levels

The levels of almost all cytokines, except IL-12, decreased with age. The most significant age-dependent inverted correlations were observed with TNF-ɑ, IL-8, IL-10, IL-4, IFN-γ, IL-5, IL-2, IL-1β, IL-13, GM-CSF, IL-21, IL-23, and IL-17A (Table 4). Concentrations of the investigated cytokines generally did not differ between genders, except for TNF-ɑ, which showed a tendency for a higher level in males compared to females (Mann–Whitney *U*-test, U = 496, p = 0.0202) in the whole study group (Table 4). We also found that IL-4, IL-6, IL-8 and IL-13 had tendency for seasonal variability (Kruskal-Wallis Rank Sum Test, p = 0.018 for IL-4, p = 0.033 for IL-6, p = 0.05 for IL-8, p = 0.019 for IL-13). The levels of IL-4 demonstrated higher values in summer compared to spring (Mann–Whitney *U*-test, U = 4, p = 0.00075) or autumn (Mann–Whitney *U*-test, U = 141, p = 0.029). The levels of IL-6 tended to be lower in spring compared to summer (Mann–Whitney *U*-test, U = 7, p = 0.0028) or winter (Mann–Whitney *U*-test, U = 24, p = 0.029). We demonstrated also that IL-8 concentration tended to increase in summer compared to spring (Mann–Whitney *U*-test, U = 8, p = 0.0041) or autumn (Mann–Whitney *U*-test, U = 144, p = 0.021), and IL-13 showed the lowest values in spring compared to summer (Mann–Whitney *U*-test, U = 4, p = 0.0020) or autumn (Mann–Whitney *U*-test, U = 71.5, p = 0.018). Other cytokines revealed no seasonal variability in our study.

**Table 4:** Cytokine associations with age and gender in the whole study group.

Cytokines	Age		Gender	
	*rho* ^#^	p^#^	U^*##*^	p^*##*^
GM-CSF	-0.47	0.0004**	422	0.3088
IL-7	-0.40	0.0026*	471	0.0610
IL-1β	-0.48	0.0003**	421	0.3171
IL-6	-0.31	0.0236*	449	0.1357
TNF-ɑ	-0.66	1.8 × 10^−7^ **	496	0.0202*
IL-8	-0.64	2.4 × 10^−7^ **	389	0.6520
IL-12	-0.26	0.0547	379.5	0.7747
IFN-γ	-0.54	2.5 × 10^−5^ **	435	0.2116
IL-2	-0.47	0.0003**	424.5	0.2860
IL-4	-0.57	6.4 × 10^−6^ **	434.5	0.2148
IL-5	-0.54	2.5 × 10^−5^ **	393	0.6028
IL-13	-0.45	0.0007**	406	0.4554
IL-17A	-0.47	0.0002**	437.5	0.4146
IL-17E	-0.31	0.0206*	382.5	0.9409
IL-17F	-0.33	0.0122*	381.5	0.9278
IL-21	-0.46	0.0004**	465.5	0.2015
IL-22	-0.36	0.0069*	394	0.9212
IL-23	-0.41	0.0017**	426.5	0.5257
IL-27	-0.35	0.0076*	393	0.9343
IL-10	-0.59	4.7 × 10^−6^ **	415	0.3700

^#^ Pairwise Spearman’s rank correlation test.

^##^ Mann–Whitney *U*-test (two-tail).

* A tendency remained after Bonferroni correction (p < 0.05 but > 0.0025).

** Statistically significant difference after Bonferroni correction (p ≤ 0.0025).

### Differences in cytokine levels between the T1D and control groups

Cytokine concentrations measured from EDTA-treated plasma samples from the control and T1D groups are reported in Table 5. The most significant difference was seen in the GM-CSF level between T1D patients and healthy controls, which remained significant even after Bonferroni correction (Mann–Whitney *U*-test, U = 169, p = 0.0018). Compared to the control group, the T1D group had tendencies (p < 0.05 but > 0.0025) towards higher concentrations of IL-7, IL-1β, IL-8/CXCL8, IL-2, the regulatory cytokine IL-10, and Th_17_ cytokines (IL-17F, IL-21, IL-23). Interestingly, the Th_1_ cytokine IL-27, but not IL-12 or IFN-γ, was upregulated in the peripheral blood of T1D patients compared to controls (Mann–Whitney *U*-test, U = 211, p = 0.011).

**Table 5:** Cytokine levels among controls and T1D patients.

Cytokine	Controls	T1D patients		
	median (IQR)	median (IQR)	U-values^#^	p-values^#^
GM-CSF	18.0 pg/ml (10.7–26.7)	35.6 pg/ml (25.0–48.9)	169	0.0018**
IL-7	2.8 pg/ml (2.0–7.2)	7.1 pg/ml (4.5–10.1)	176	0.0034*
IL-1β	3.2 pg/ml (1.6–5.6)	6.4 pg/ml (4.2–8.5)	198	0.0104*
IL-6	2.6 pg/ml (1.7–6.9)	4.8 pg/ml (2.7–9.1)	239	0.0718
TNF-ɑ	8.0 pg/ml (5.0–17.1)	11.9 pg/ml (9.1–14.1)	271	0.2220
IL-8	4.8 pg/ml (3.0–6.1)	6.5 pg/ml (4.4–9.6)	219.5	0.0352*
IL-12p70	4.3 pg/ml (2.4–12.9)	4.9 pg/ml (3.2–8.4)	311	0.6097
IFN-γ	4.8 pg/ml (3.1–12.6)	8.9 pg/ml (6.5–15.0)	235.5	0.0625
IL-2	4.2 pg/ml (2.8–6.3)	7.6 pg/ml (5.2–9.7)	193	0.0087*
IL-4	8.8 pg/ml (4.7–15.5)	14.0 pg/ml (7.7–25.7)	253	0.1212
IL-5	0.9 pg/ml (0.2–2.1)	0.8 pg/ml (0.6–1.6)	306	0.5485
IL-13	2.3 pg/ml (0.5–13.5)	4.7 pg/ml (2.2–8.7)	265	0.1817
IL-17A	36.1 pg/ml (21.0–65.0)	64.6 pg/ml (34.5–83.8)	246	0.0523
IL-17E	0.9 ng/ml (0.5–1.5)	1.3 ng/ml (1.0–1.7)	250.5	0.0623
IL-17F	0.03 ng/ml (0.01–0.04)	0.04 ng/ml (0.03–0.06)	240.5	0.0418*
IL-21	62.6 pg/ml (41.8–79.1)	101.6 pg/ml (70.1–125.3)	215.5	0.0138*
IL-22	0.3 ng/ml (0.2–0.5)	0.4 ng/ml (0.3–0.6)	265	0.1061
IL-23	2.4 ng/ml (1.7–3.7)	3.9 ng/ml (2.8–5.3)	232	0.0292*
IL-27	1.3 ng/ml (1.0–1.7)	1.8 ng/ml (1.5–2.2)	211	0.0111*
IL-10	16.8 pg/ml (9.4–26.6)	25.2 pg/ml (18.9–31.6)	203	0.0136*

^#^ Differences in cytokine levels between controls and T1D patients. The Mann–Whitney *U*-test (two-tail) was used to calculate significant differences.

* A tendency remained after the Bonferroni correction (p < 0.05 but > 0.0025).

** Statistically significant difference after the Bonferroni correction (p ≤ 0.0025).

### Influence of blood sampling time and diabetic ketoacidosis (DKA) on cytokine levels in the T1D group

**Fig 1:**
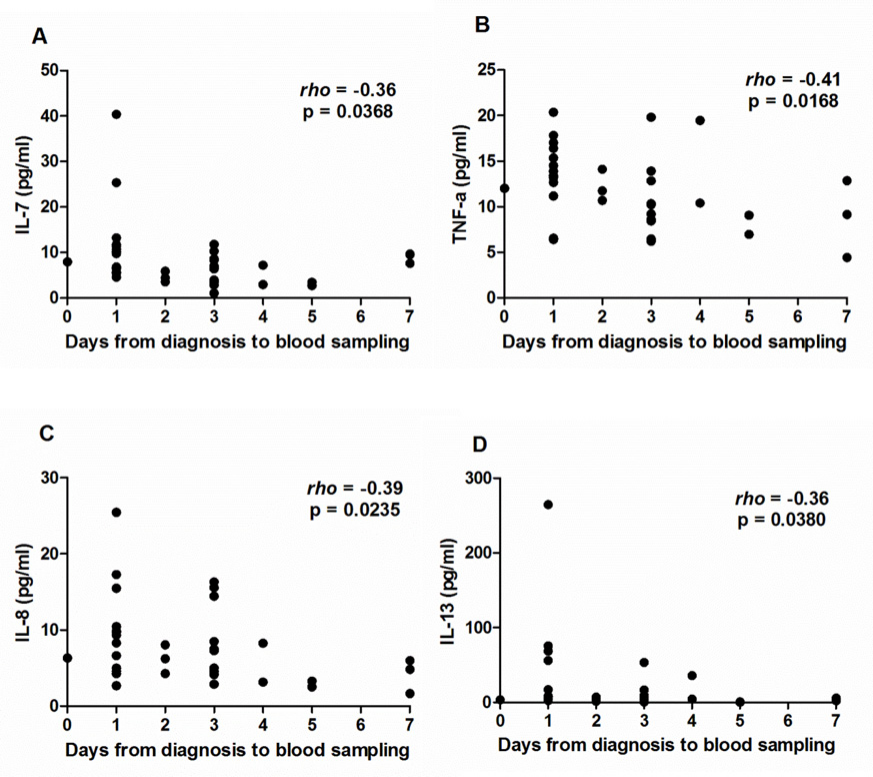
Association of time between T1D diagnosis and blood sampling with cytokine levels. After the Bonferroni correction, the levels of IL-7 (A), TNF-ɑ (B), IL-8 (C), and IL-13 (D) showed a tendency to decrease with an increasing number of days between T1D diagnosis and blood sampling. Spearman’s rank correlation test was used.

**Fig 2:**
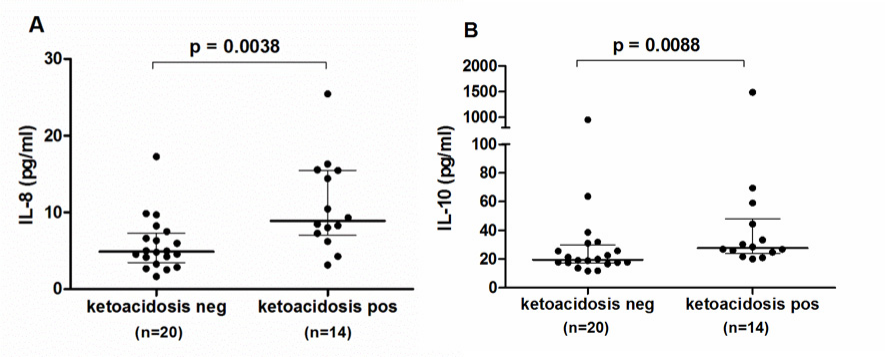
Differences in cytokine levels between T1D patients with and without ketoacidosis. Compared to T1D patients without ketoacidosis, patients with ketoacidosis had a tendency for higher IL-8 (A) and IL-10 levels after Bonferroni correction* (B). Lines represent median and interquartile range values. * P-values ≤ 0.0025 were considered statistically significant after Bonferroni correction in Mann-Whitney U-test.

Almost half (44%) of investigated children suffered from DKA at the time of T1D diagnosis. As accompanying metabolic imbalance and insulin treatment could influence the production of several cytokines, we analyzed whether the time since diagnosis affected the cytokine concentrations. The IL-7, TNF-ɑ, IL-8, and IL-13 levels showed a tendency (p < 0.05 but > 0.0025) to decrease with an increasing time gap between diagnosis and blood sampling (Fig 1). Moreover, T1D patients with DKA had a tendency for higher IL-8 (Mann–Whitney *U*-test, U = 59, p = 0.0038) and IL-10 levels (Mann–Whitney *U*-test, U = 66, p = 0.0088) compared to T1D patients without DKA (Fig 2).

### Diabetes-related autoantibodies and cytokine levels

Most of the T1D patients (Table 1) had more than one diabetes-specific autoantibody: 36% had two and 53% had three. Only two T1D patients, neither of whom had DKA, had no autoantibodies. Two other patients with T1D, one with and one without DKA, had only one autoantibody, which was GADA in both cases.

In the control group, two individuals had low titers of GADA and one individual had a low titer of ZnT8A. All of these autoantibody-positive controls were classified in the low-risk group on the basis of HLA haplotype. Different laboratories, including ours, have reported low titers of diabetes-associated antibodies among healthy persons [19–21].

**Fig 3:**
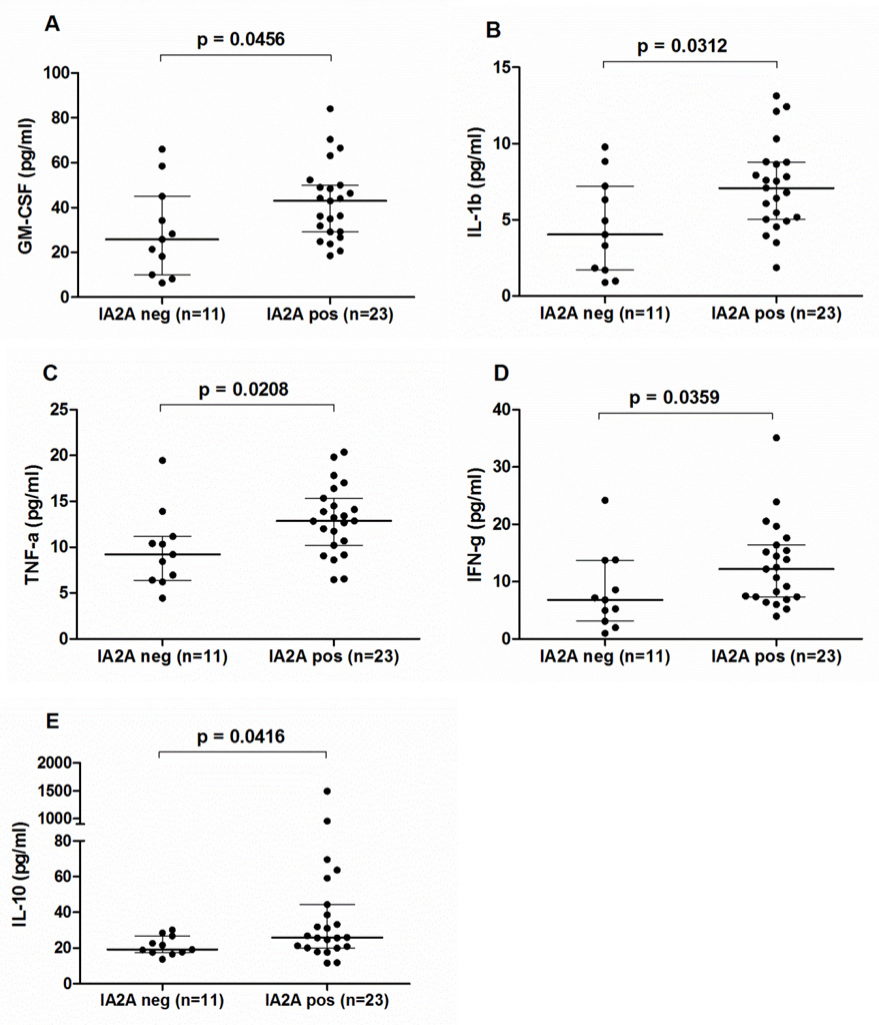
Differences in cytokine levels between T1D patients with and without IA2A. Compared to T1D patients without IA2A, T1D patients with IA2A showed a tendency for higher GM-CSF (**A**), IL-1β (**B**), TNF-ɑ (**C**), IFN-γ (**D**), and IL-10 (**E**) levels Bonferroni correction^*^. Lines represent median and interquartile range values. ^*^ P-values ≤ 0.0025 were considered statistically significant after Bonferroni correction in Mann–Whitney *U*-test.

**Fig 4:**
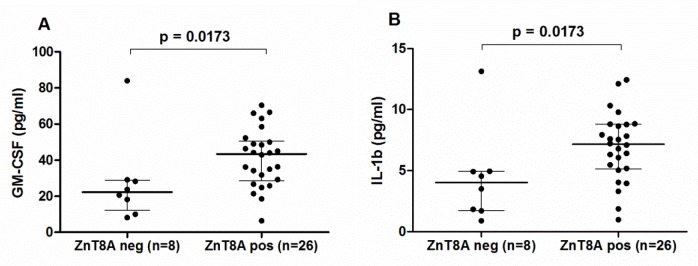
Differences in cytokine levels between T1D patients with and without ZnT8A. Compared to T1D patients without ZnT8A, T1D patients with ZnT8A showed a tendency for higher GM-CSF (A) and IL-1β (B) levels after Bonferroni correction*. Lines represent median and interquartile range values. * P-values 0.05–0.0025 were considered as a tendency after Bonferroni correction in Mann–Whitney U-test.

There was no significant difference in cytokine levels among T1D patients with and without GADA. However, the IA2A-positive T1D group showed a tendency towards higher IL-1β (Mann–Whitney *U*-test, U = 68, p = 0.0312), TNF-ɑ (U = 64, p = 0.0208), IFN-γ (U = 69, p = 0.0359), GM-CSF (U = 72, p = 0.0456), and IL-10 (p = 0.0416) levels compared to IA2A-negative patients (Fig 3). Moreover, T1D patients with ZnT8A revealed a tendency for higher IL-1β and GM-CSF levels (Mann–Whitney *U*-test, U = 46, p = 0.0173 for both) compared to ZnT8A-negative patients (Fig 4). In addition, we analyzed association of cytokine levels and multiple autoantibodies in blood. It was revealed that the level of IL-1β, IFNγ and GM-CSF was higher in T1D patients with two or more autoantibodies compared to the patients with single autoantibody or without any (Mann–Whitney *U*-test; U = 12.36, p = 0.00044 for IL-1β; U = 6.58, p = 0.010 for IFN-γ and U = 6.86, p = 0.0088 for GM-CSF).

### Diabetes-related HLA haplotypes and cytokine levels

Most of the examined T1D patients were categorized as having a moderate or high risk for T1D (28% and 25%, respectively) on the basis of HLA haplotypes (Table 1). Seven T1D patients (19%) were categorized as having a decreased risk. In the control group, only two individuals (10%) were classified as being HLA risk for T1D. Besides, we detected no statistically significant differences or tendencies in cytokine levels between T1D patients in the high-risk HLA group compared to T1D patients in all other risk groups (data not shown).

## Discussion

In general, T1D is considered to be a Th1-type autoimmune disease caused by pancreatic attack by autoreactive T cells. Various inflammatory cells producing different proinflammatory cytokines could also be involved, and pancreatic β-cell destruction accompanies the inflammatory response (insulitis) within the islets [22]. Destructive insulitis is associated with elevated levels of Th1 cytokines (IL-2, IL-12, and IFN-γ) and proinflammatory cytokines (IL-1β, IL-6, TNF-β, and IFN-α) in animal models [1,23]. However, despite evidence for the upregulation of several of the aforementioned cytokines at [1,24] and before [4,25] T1D onset in humans, the published results are inconsistent across studies [3].

In our study population, we demonstrated the differences in proinflammatory cytokine (IL-1β and IL-8) levels between young newly diagnosed T1D patients compared to age-matched healthy controls. The highest level of proinflammatory cytokines was observed in patients exhibiting IA-2 and Zn-T8 autoantibodies. These signs of activation of the innate immune system are partly consistent with reports of IFN-α/γ and IL-1β pathway activation associated with altered Toll-like receptor responsiveness and enhanced nuclear factor (NF)-κB signaling in the dendritic cells (DCs) and monocytes of newly diagnosed T1D patients [9,26]. However, in contrast to the decreased IL-6 levels reported in these previous publications, we found no changes in IL-6 or TNF-ɑ levels in our T1D patients with young age of onset.

In terms of pathogenesis, we did not detect an obvious imbalance of Th_1_/Th_2_ polarization towards prominent activation of Th_1_. A recent publication showed that a dominant Th_1_-associated immune profile in the prediabetic phase could switch to a Th_3_-associated profile, with a burst of inflammatory cytokines, immediately before clinical onset of T1D [27]. These results suggest the consequences of an imbalance of the innate immune system, which would trigger islet disturbances and apoptosis that, in turn, could lead to clinical onset of the disease. Metabolic disorders could complicate the early clinical situation of T1D to which implies a systemic elevation of IL-8 in T1D patients with DKA in our study. Several ongoing clinical trials are investigating the effect of proinflammatory cytokine blockade in subjects with recent-onset T1D, demonstrating the interest in regulating the innate immune system in this disease [28].

Perhaps the most surprising and statistically significant (p < 0.005) result of the current study was the upregulation of the growth factors GM-CSF and IL-7 in the peripheral blood of T1D patients. Although GM-CSF is best known for its role in myeloid differentiation, it is also a potent growth factor for monocytes, macrophages, and DCs. Previous reports have proposed that the increased GM-CSF levels in nonobese diabetic (NOD) mice and T1D patients may represent the organisms’ efforts to compensate for the defective responses of the hematopoietic cells (including bone marrow-derived DCs and pancreatic macrophages) to this growth factor [29,30]. Supporting this suggestion, recent studies have demonstrated that GM-CSF/IL-3–deficient mice develop insulitis, precipitated by the administration of anti–CTLA-4 blocking antibodies, with destruction of insulin-producing β-cells and compromised glucose homeostasis [31]. Defects in the phagocytosis of apoptotic cells by macrophages might contribute to autoimmune diabetes by decreasing the production of immunoregulatory cytokines and increasing the production of proinflammatory cytokines [32]. However, we observed the joint elevation of GM-CSF and IL-10 in the blood of T1D patients, which may reflect the activation of protective immune mechanisms. Repeated treatment of NOD mice with GM-CSF was shown to prevent the development of insulitis by inducing tolerogenic DCs, which sustained the persistent suppressive function of IL-10–producing T_reg_ cells [33]. Interestingly, GM-CSF was more effective when administered at later stages of insulitis. Similarly, the therapeutic potential of GM-CSF in human T1D could be speculated [34].

IL-7 is a major homeostatic cytokine for several cell types of the immune system. Overexpression of this growth factor or its receptor has been associated with the severity of autoimmune disease in animal models [35] and humans [36,37]. A study proposed that the provision of exogenous or lymphopenia-induced endogenous IL-7 promotes the expansion of self-reactive clones, even in the presence of T_reg_ cells, thereby explaining the relevance of IL-7 in the development of diabetes [38]. However, a recent study reported the existence of diabetes-suppressive IL-17–expressing DCs that were capable of promoting the maturation of IL-7–responsive CD^25+^ CD^127+^ T_reg_ cells [39]. In these T cells, IL-7 maintains the expression of FoxP3 and CTLA4, which could represent an additional non-IL2–dependent compensatory mechanism for T_reg_ cell survival and functional activity.

We discovered an increased level of IL-27 in the peripheral blood of newly diagnosed T1D patients compared to healthy individuals. To our knowledge, this is the first study demonstrating the up-regulation of IL-27 in human T1D. Previously, this phenomenon has been described in granulomatous diseases [40], inflammatory bowel disease [41], and multiple sclerosis [42]. IL-27 is a pleiotropic cytokine of the IL-12 family with both inhibitory and activating functions on innate and acquired immunity. IL-27 is secreted by activated antigen presenting cells (macrophages, DCs) and demonstrates inhibitory effects on the development of Th_1_, Th_2,_ and Th_17_ cells, as well as on the expansion of T_reg_ (reviewed in [43]).

The role of IL-27 in autoimmune diabetes has been insufficiently investigated and the results obtained have been inconsistent. Blockade of IL-27 delays the onset of diabetes in NOD mice [44]. However, it was recently reported that IL-27 can inhibit streptozotocin-induced hyperglycemia and pancreatic islet inflammation in an animal model and therefore could represents a potential novel therapeutic approach for T1D [45]. In humans, the association of IL-27 polymorphisms with T1D has been reported in genome-wide association studies [46], but these results were not confirmed by others [47].

IL-27 suppresses effector Th_17_ cells and promotes the generation of type 1 regulatory T (Tr1) cells, which, in turn, could dampen autoimmunity and tissue inflammation by secreting the immunosuppressive cytokine IL-10 [48]. We found that the IL-27 level correlated very strongly (*rho* > 0.9) with the levels of several Th_17_ cytokines, but relatively weakly with IL-10 levels (*rho* = 0.39). Moreover, the Th_17_ cytokines IL-17A, IL-21, and IL-23 and the regulatory cytokine IL-10 demonstrated notable upregulation in the T1D group compared to the control group. Taken together, these findings could support the hypothesis that several regulatory mechanisms, in particular those acting via IL-27 and IL-10, attempt unsuccessfully to dampen the harmful effects of Th_17_ immunity in T1D patients. Not only Th_1_, but also Th_17_ cells and hyperfunction of proinflammatory cytokines may play detrimental roles at the onset and during metabolic decompensation in recent-onset young-age T1D [1,49].

Limitations of this study include the assessment of cytokine concentrations in plasma alone. As peripheral blood cells from the current study groups were not available to us, we could not detect the spontaneous or stimulated production of cytokines by distinct immune cells. However, we believe that careful profiling of circulating cytokines in blood provides valuable data about the (dys)regulation of the immune system *in vivo*. The peripheral blood is arguably more appropriate for clinical purposes, especially for large cohorts with limited volumes of material (e.g., from children).

We stress the importance of the correct preparation and storage of plasma samples, as well as the avoidance of freeze/thaw cycles before the cytokines are studied. For some biomarkers, the measured level may either decrease or increase several times after repeated freeze/thaw cycles [50]. Another strength of this study was the ability to measure multiple cytokines simultaneously with the same small amount of probe by a highly sensitive method. This approach is an important element for the correct measurement of low-concentration cytokines in human serum, which often require highly sensitive assays for detection. The careful selection of the target patient population was also important, because the variability of insulin treatment length and the concurrence of other autoimmune/inflammatory diseases may significantly affect the results. The same consideration also applies to the choice of control children, who are the most difficult study group in humans.

### Conclusions

Our findings broaden the current understanding of the dysregulation of systemic cytokine levels at the onset of young-age T1D. This dysregulation includes the upregulation of growth factors (GM-CSF and IL-7) and proinflammatory factors (IL-1β but not IL-6 or TNF-β) of the innate immune system, as well as Th_17_ cytokines and regulatory cytokines (IL-10, IL-27).
